# A case of hemorrhage at the junctions of the posterior intercostal arteries—a vital sign?

**DOI:** 10.1007/s00414-024-03261-9

**Published:** 2024-06-11

**Authors:** Nicolas Lange-Herr, Joëlle Tschui, Jeremias Klaus, Manuela Baglivo, Emilie Schlottke, Wolf-Dieter Zech

**Affiliations:** 1 Institute of Forensic Medicine, University of Bern, Bern, Switzerland; 2 Medics Pathologie AG, Bern, Switzerland; 3 Department of Diagnostic, Interventional and Pediatric Radiology, Inselspital, Bern University Hospital, University of Bern, Bern, Switzerland; 4 Dr. Kurz Röntgeninstitut AG, Thun, Switzerland; 5 University of Zurich, Zurich, Switzerland

**Keywords:** Hanging, Suicide, Simon’s hemorrhage, Vital signs

## Abstract

The authors present the case of a 58-year-old man found hanging from a radiator by his shoelaces. The time of death was approximately 6 h before the body was discovered. An autopsy was performed approximately 24 h after the body was found, which revealed hemorrhages in the thoracic aorta at the junctions of the posterior intercostal arteries. Before autopsy, a routine whole-body CT scan was performed. Histologic examination of the aorta and the posterior intercostal arteries revealed a fresh hemorrhage into the tunica adventitia of the aorta. To our knowledge, there is no case description of such findings in hanged persons in the literature. Conclusion: Hemorrhages into the tunica adventitia of the junction of the posterior costal arteries may occur in association with suicidal hanging. The significance of these hemorrhages as a sign of vitality may be debated.

## Case circumstances

A 58-year-old man was found hanged at his residence. Doors and windows were locked, and there was no evidence of a fight or argument. No suicide note, drugs, or medication were found at the scene. The police investigation revealed no evidence of physical or mental illness. In particular, the investigation revealed no evidence of anticoagulant medication, pre-existing blood clotting disorders, or vascular diseases. On examination, a cylindrical radiator, approx. 2.2 m high and approx. 20 cm in diameter showed a pin-like protrusion on the top. Around this pin-like protrusion was a ligature consisting of several partially interconnected laces of different colors. The person hung freely in the ligature, without contact with the ground. The ligature ran in a circle around the neck and nape of the neck and rose on both sides of the neck toward the nape. The highest point was in the middle of the neck. This corresponds to a typical hanging. The ligature length from the highest point in the neck of the deceased to the attachment to the heating element was approx. 25 cm. Next to the body was an overturned dustbin. There were whitish, dry adhesions on the lower lip on the left side and the front of the T-shirt consistent with saliva stains. When the body was found, there were clear signs of death and no resuscitation measures were initiated. Based on the findings and forensic examinations, the time of death was estimated to be approximately 6 h before the body was found.

## Post-mortem findings

Prior to the autopsy, we conducted a routine whole-body unenhanced CT scan. The interval between death and PMCT was approximately 30 h. Postmortem CT examination revealed no fresh bone fractures and no forensically relevant foreign bodies or gas accumulations. An autopsy was performed immediately after imaging.

## Autopsy and histology

The autopsy was performed immediately after the CT examination by forensically certified pathologists in accordance with the Recommendation of the Committee of Ministers to the Member States of the European Union on the Harmonization of Rules for Forensic Autopsy [1] and therefore included an external and internal examination with opening of all three body cavities and dissection of all organs. During the autopsy, tissue samples of the aorta and tissue samples of various other internal organs were collected for histological examination. Hematoxylin and eosin (H&E) and Elastin van Gieson (EVG) were used as histological stains. A certified forensic pathologist made histological examinations and diagnosis. The prosecutor’s office did not order toxicological analyses.

**Fig. 1:**
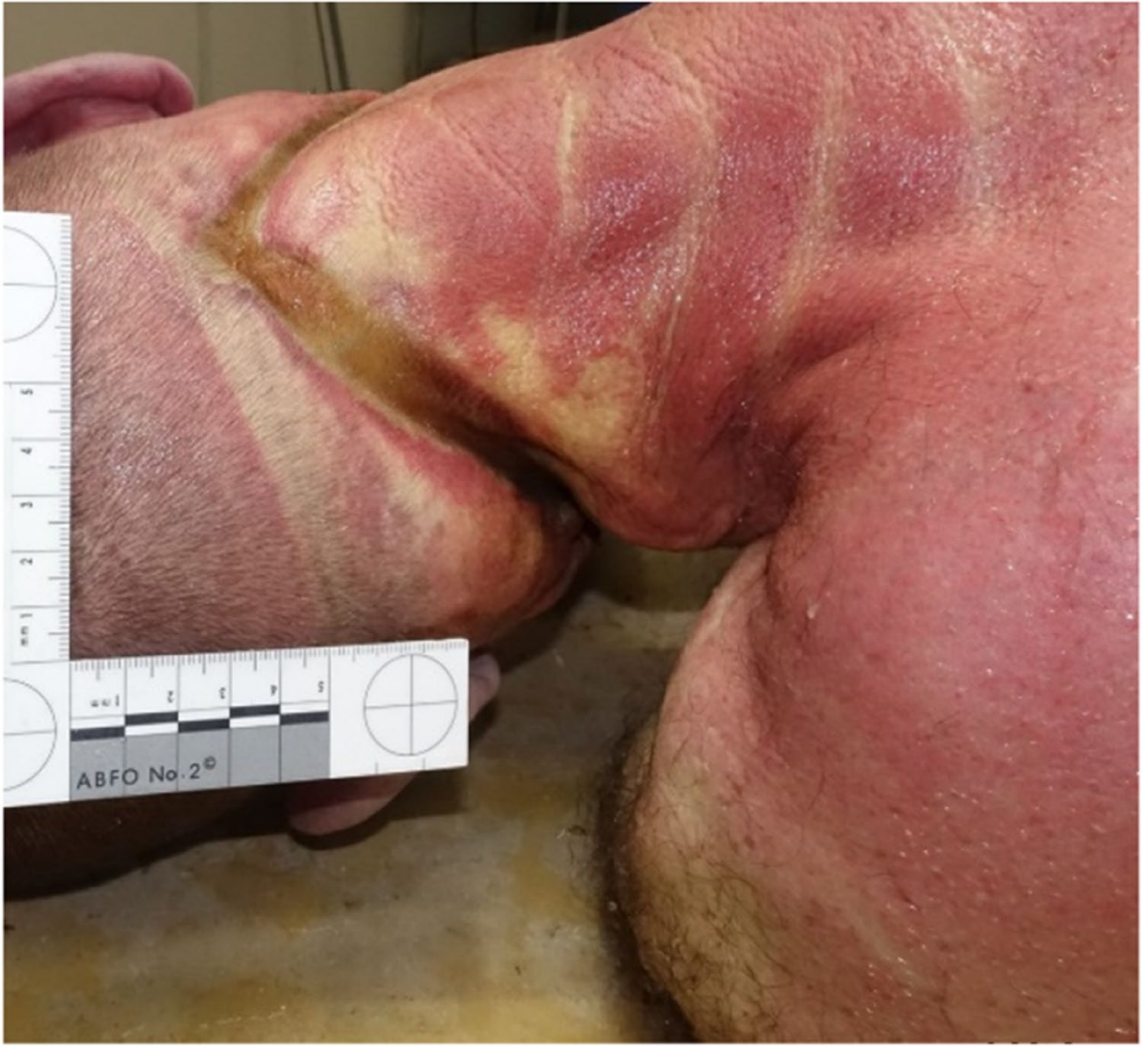
Circular furrow around the neck. View from the back

The external examination showed a brownish strand furrow running circularly around the neck and nape, rising on both sides of the neck toward the nape, about 1.5 cm wide (Fig. 1), patchy skin desiccation on the left cheek, superficial skin breakdown on the left thumb, and multiple patchy bruises on the lower extremities. The torso showed no fresh injuries that would indicate blunt force trauma.

**Fig. 2:**
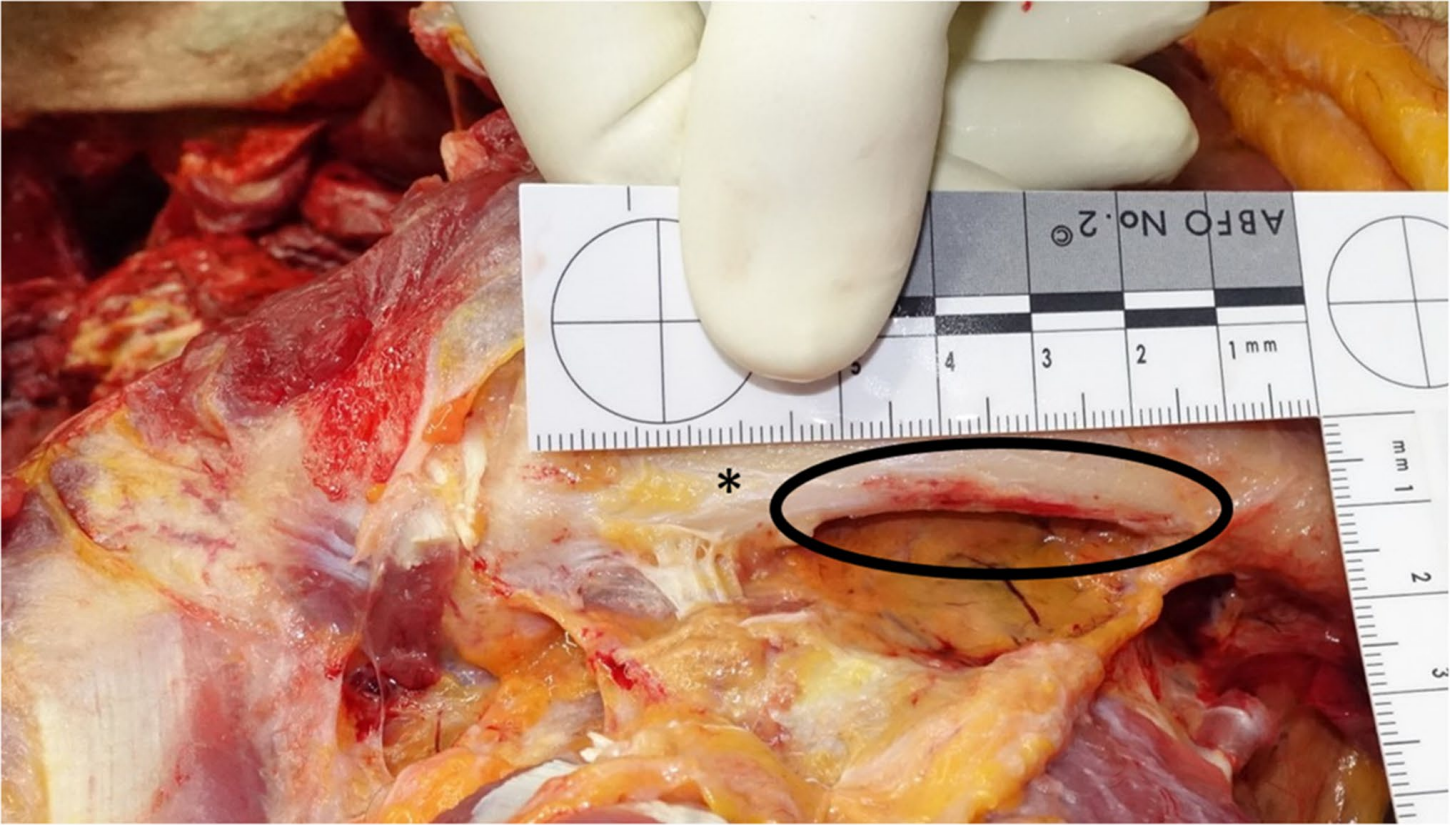
Periosteal hemorrhages (circle) on the upper surfaces of the clavicle (asterisk)

**Fig. 3:**
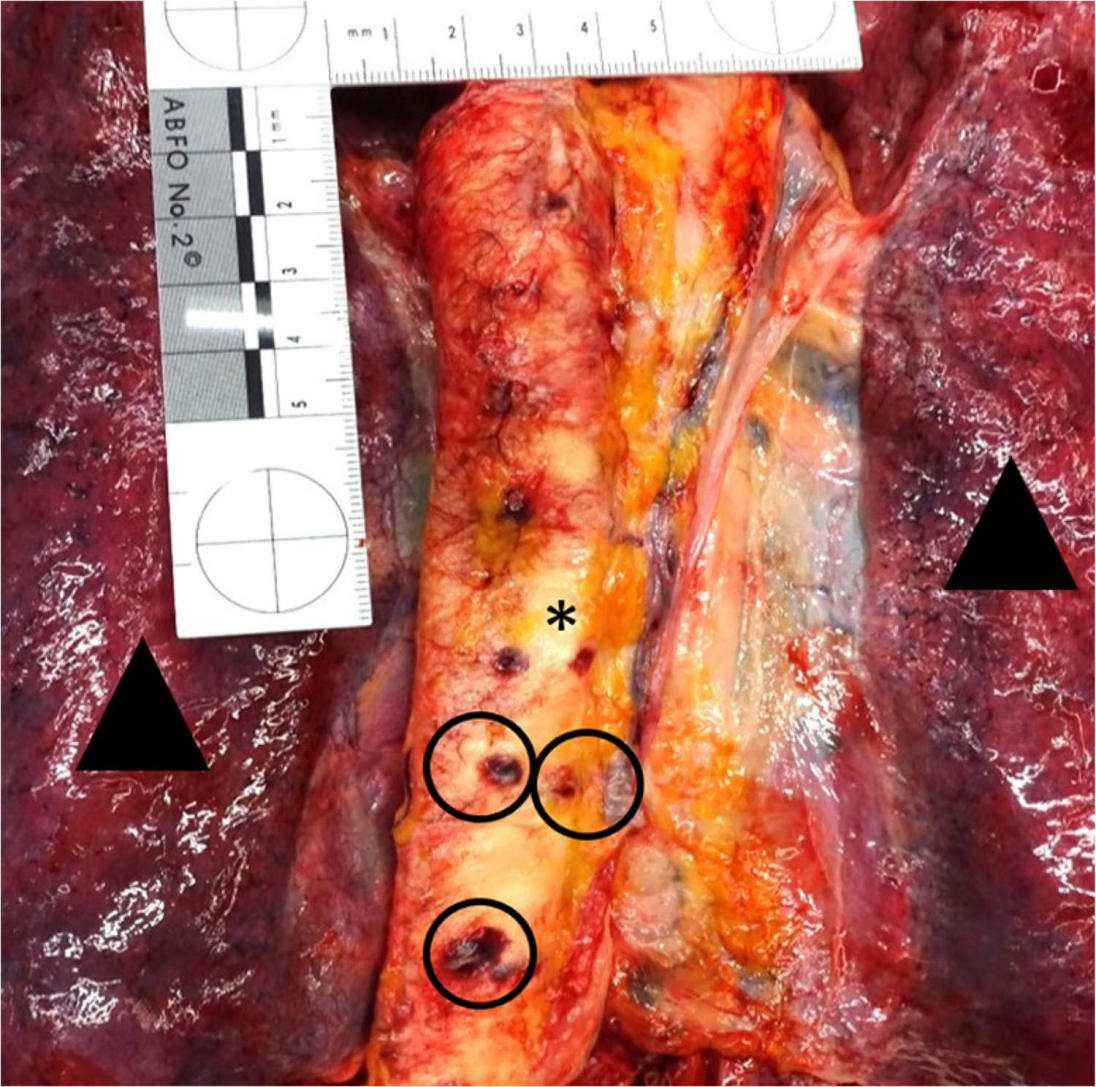
Hemorrhages (circles) in the tunica adventitia of the thoracic aorta (asterisk) at the origin of the posterior intercostal arteries. Arrowheads: right and left lung

Internal examination including the soft tissue dissection of the back revealed a congested scalp with multiple punctate hemorrhages, congested cerebral medulla and cerebral edema, extensive periosteal hemorrhages on the upper surfaces of both clavicles (Fig. 2), multiple transverse tears of the intima of the left carotid artery, fracture of both superior horns of the thyroid cartilage with hemorrhages into the surrounding soft tissues, hemorrhages into the soft tissues surrounding the hyoid bone, patchy hemorrhages in the right infraspinatus and the left supraspinatus muscle, and hemorrhages in the tunica adventitia of the thoracic aorta at the origin of the posterior costal arteries (Fig. 3). No evidence of blunt force trauma, Simon’s bleedings, further vascular dissection, or evidence of disease contributing to vascular dissection or other forensically relevant conditions was found.

**Fig. 4:**
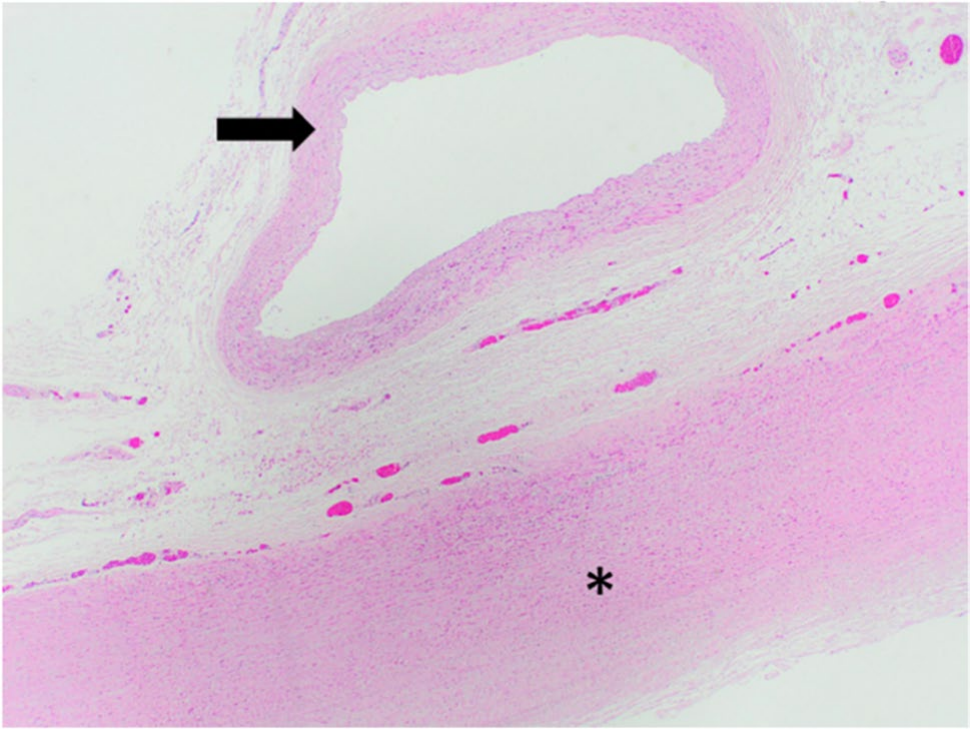
Asterisk: Aorta, Arrow: A. intercostales posterior. Hematoxylin–eosin staining × 2

**Fig. 5:**
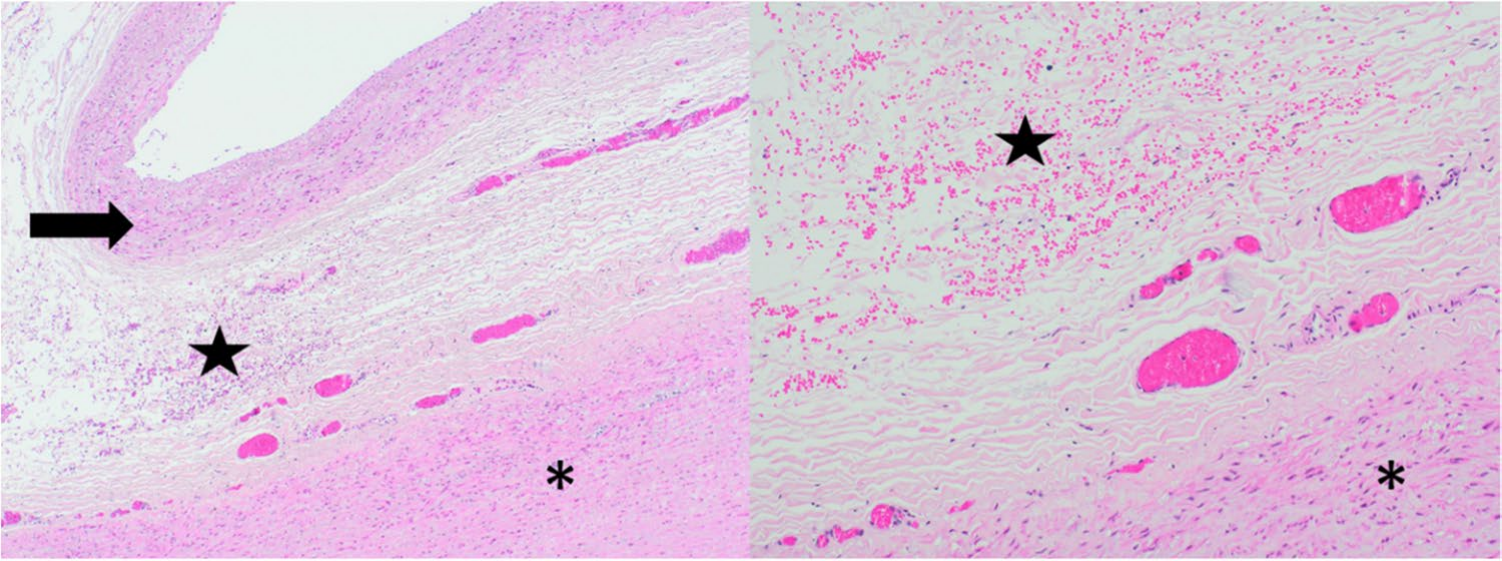
Asterisk: Aorta, Arrow: A. intercostales posterior, Star: fresh hemorrhage into the tunica adventitia. Hematoxylin–eosin staining, left × 4, right × 10

H&E and EVG staining at the origin of the posterior costal arteries showed fresh hemorrhage into the tunica adventitia of the aorta (Figs. 4 and 5).

The manner of death was not natural and the findings are compatible with the assumption of suicide. The cause of death was in the absence of competing causes of death and subject to toxicological tests not having been carried out determined to be a central regulatory failure due to lack of oxygen to the brain as a result of compression of the neck vessels.

## Discussion

Suicides are a common occurrence in the work of a forensic pathologist. According to eurostat, there were more than 47,000 suicides registered in the European Union in 2020 [2]. Although there is no information about the number of those hanged, it can be assumed that it is significant. For example, in Germany in 2020, the hanging method was used in more than 45% of suicides [3].

In some of these cases, the responsible public prosecutor’s office orders further examinations, such as an autopsy. The findings in the present case (periosteal hemorrhages on the upper surfaces of both clavicles, tears of the intima of the carotid arteries, fractures of the laryngeal skeleton, hemorrhages in the soft tissue of the neck and pharyngeal region, hemorrhages in the muscles of the back) are typical in people who have died by hanging and have been published for many years [4–10]. Other typical findings in death by hanging, such as Simon’s bleeding [11], could not be visualized in this case. Unexpected findings always require a thorough examination and a possible mechanism must be discussed, among other things to rule out a possible external influence [12]. The congested scalp is not a typical finding in the context of a typical suspension and other indications of a congestion syndrome in the sense of point bleeding in the mucous membranes could not be demonstrated. One possible mechanism would be an initially slow descent into the loop. Our literature search regarding hemorrhages in the junctions of the posterior intercostal arteries did not reveal any description in connection with hanging. Hemorrhages and especially injuries of the posterior intercostal arteries are described in the literature, however mostly in connection with fractures of ribs or vertebrae as a result of blunt force trauma [13, 14]. As already mentioned at the above, there was no evidence of blunt force trauma in the case presented here. In current literature, bowel wall hemorrhage is discussed as a sign of death by hanging [15, 16]. The underlying mechanism is thought to be abdominal congestion, particularly in cases of prolonged agony [16]. Nikolić et al. analyzed a total of 647 cases to determine the frequency of Simon’s hemorrhage in hanging and other asphyxiation cases [11]. The postulated mechanism of Simon’s hemorrhage is considered hyperextension of the spine due to gravity and agonal spasms of the subject. Schulz et al. [7] describe bleeding into the back muscles in connection with deaths by hanging. They postulate maximal inhalation attempts as a possible mechanism in addition to convulsions. A similar mechanism for the development of hemorrhages in the insertions of the posterior intercostal arteries can be discussed. However, Schulz et al. did not describe any such hemorrhages in their cases, and the development of hemorrhages due to breathing seems less likely. The authors of the present case report hypothesize a similar mechanism in the development of the hemorrhages described here.

## Conclusions

If the mechanism of origin of these hemorrhages is equivalent to that of Simon’s hemorrhage, it can be assumed that these hemorrhages are also to be regarded as vital signs. The fact that, to the authors’ knowledge, this is the first description of these hemorrhages in connection with hanging suggests that such hemorrhages occur only in rare cases. Nevertheless, the correct classification of these findings can support the thesis of a vital hanging in case of doubt. Hemorrhages into the tunica adventitia of the junction of the posterior costal arteries may occur in association with hanging. The significance of these hemorrhages as a sign of vitality may be debated.
